# The relationship between alcohol consumption and health: J-shaped or less is more?

**DOI:** 10.1186/s12916-023-02911-w

**Published:** 2023-07-03

**Authors:** Min-Kuang Tsai, Wayne Gao, Chi-Pang Wen

**Affiliations:** 1 Program in Global Health and Health Security, College of Public Health, Taipei Medical University, 250 Wuxing Street, 110 Taipei, Taiwan; 2 Institute of Population Health Sciences, National Health Research Institutes, Miaoli, Taiwan; 3 China Medical University Hospital, Taichung, Taiwan

**Keywords:** Alcohol consumption, J-shaped relationship, Risk factor, Disease burden, Mortality, Cohort study

## Background

In this issue of the Journal, Tian and colleagues confirmed the J-shaped relationship between alcohol consumption and health based on analyzing 918,529 adults from the repeated National Health Interview Survey (NHIS) from 1997 to 2014 [1]. In addition to several cause-specific mortality, Tian and colleagues’ findings are largely confirmatory to previous studies using NHIS [2, 3] and several large systematic review and meta-analysis on this issue [4–6].

The “J-shaped” relationship refers to a protective health effect at a lower level of consumption; while above a certain threshold, it greatly increases health risks. This study found that compared with lifetime abstainers, current infrequent, light, and moderate drinkers had a lower risk of all-cause mortality (current infrequent: − 13%; light: − 23%; moderate: − 18%) and cardio-vascular disease mortality (infrequent: − 14%; light: − 24%; moderate: − 22%), respectively. However, it is important to address several methodological issues when interpreting the J-shaped association.

## Main text

Firstly, the issue of misclassification must be considered, which pertains to the exclusion of “sick quitters” or the “abstainer bias” from the definition of the reference group [7]. In order to mitigate this error, some studies have attempted to define the reference group as lifetime abstainers, while excluding ex-drinkers, as done by Tian et al. [1]. However, also using lifetime nondrinkers as reference, an updated systematic review and meta-analysis of 107 cohort studies with near half million participants concluded that the low-volume alcohol drinking was not associated with protection against death from all causes [8].

Secondly, other likely biases included the potentials for reverse causation, a possibility that individuals who are already in poor health may be more likely to be abstainers and individuals with high health conscious and in good shape also tends to be more reasonable drinkers, which could lead to better health. In addition, in the Western societies where most J-shape studies derived from, it is possible that light to moderate drinking may be associated with active social and family life, higher socioeconomic status, healthier lifestyle choices, and access to better healthcare, which may independently contribute to better health outcomes.

Thirdly, alcohol can be addictive, and even moderate consumption can lead to dependence and addiction in susceptible individuals. Therefore, promoting even moderate alcohol consumption based on a J-shaped relationship may send mixed messages and result in increased alcohol consumption, leading to negative population health outcomes.

Fourthly, it seems that when determining what constitutes as an optimal level of alcohol consumption, in epidemiological terms, theoretical minimal exposure level (THML) varies significantly among individuals and disease burden across different regions [9, 10]. For instance, for young population age, it is close to zero consumption while the potential beneficial effect particularly with respect to cardiovascular disease maybe more salient in older age groups and geographic locations where CVD burden are high.

Fifthly, the standard of alcohol consumption is also challenging for drinkers to adhere to, and the impact of different drinking amounts on average life expectancy has been studied previously [11]. The findings revealed that the benefits of moderate drinking were negated by a two to fourfold increase in oral and esophageal cancer risk, and excessive drinking resulted in a significant reduction in life expectancy. Therefore, the threshold for safe alcohol consumption is often ambiguous.

It is challenging to tease out the direct effects of alcohol consumption from these biases and confounding factors in observational studies. However, these inconsistent findings may be resolved by some methodological advancement. To address causal questions in observational studies, Mendelian randomization has been used, and it was found that it is not possible to draw conclusion on the causal role of moderate drinking and cardio-metabolic health [12]. The same conclusion has been drawn from a genetic epidemiological study which shows that the apparently protective effects of moderate alcohol intake against stroke are largely non-causal [13]. This year (2023), the World Health Organization (WHO) published a statement indicating that “when it comes to alcohol consumption, there is no safe amount that does not affect health” [14] and alcohol is responsible for 3 million deaths in 2016 globally and 5.1% of the global burden of disease and injury estimated by the WHO [15]. The updated guidance on alcohol health in Canada also presented a continuum of risk and stated that “Drinking less is better” [16].

## Conclusion

**Fig. 1:**
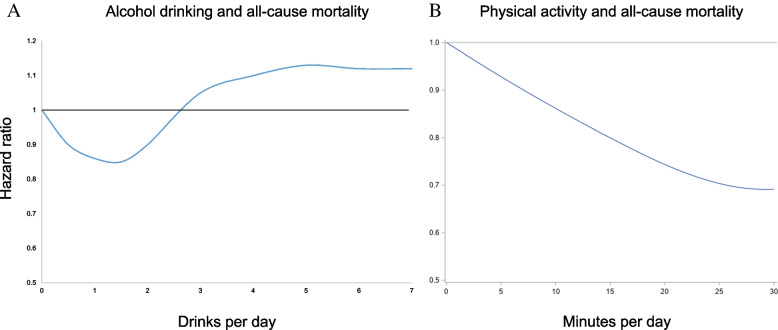
Relative risk curve of physical activity in comparison to a disputed typical J-shaped risk for alcohol drinking. Individuals who engage in 15–20 min of physical activity per day experience a similar level of reduced mortality as in the debated J-shaped association between moderate drinking and health. Figure 1A data source is from Tian et al. study [1]. Figure 1B data source is from our previous cohort study [17]

In conclusion, while some studies have suggested a J-shaped relationship, there are limitations in the study design, confounding factors, and individual variability that challenge the generalizability and interpretation of these findings. More recent studies with advanced methodological designs have challenged the J-shape association. It is also important to consider societal/population-level harmful effects of alcohol use and alternative health-promoting strategies when evaluating the implications of the J-shaped relationship. Here, we provide relative risk curve of physical activity in comparison to a debated typical J-shaped for life-time abstainer (Fig. 1). In contrast to less is more in alcohol use; many alternative healthy lifestyle choices mean more is merrier.

## Data Availability

The MJ Health Research Foundation administered MJ Health Survey Database and MJ BioData. Data will be shared on request to the corresponding author with permission of MJ Health Research Foundation.
